# Persistent cryoglobulinemia after antiviral treatment is associated with advanced fibrosis in chronic hepatitis C patients

**DOI:** 10.1371/journal.pone.0268180

**Published:** 2022-05-13

**Authors:** Batbold Batsaikhan, Ching-I. Huang, Ming-Lun Yeh, Chung-Feng Huang, Yi-Hung Lin, Po-Cheng Liang, Ming-Yen Hsieh, Yi-Ching Lin, Jee-Fu Huang, Wan-Long Chuang, Jin-Ching Lee, Ming-Lung Yu, Hsing-Tao Kuo, Chia-Yen Dai

**Affiliations:** 1 Department of Internal Medicine, Institute of Medical Sciences, Mongolian National University of Medical Sciences, Ulaanbaatar, Mongolia; 2 Graduate Institute of Medicine, College of Medicine, Kaohsiung Medical University, Kaohsiung, Taiwan; 3 Hepatobiliary Section, Department of Internal Medicine, Kaohsiung Medical University Hospital, Kaohsiung Medical University, Kaohsiung, Taiwan; 4 School of Medicine, College of Medicine, Kaohsiung Medical University, Kaohsiung, Taiwan; 5 Department of Internal Medicine, Kaohsiung Municipal Hsiao-Kang Hospital, Kaohsiung Medical University, Kaohsiung, Taiwan; 6 Department of Internal Medicine, Kaohsiung Municipal Ta-Tung Hospital, Kaohsiung Medical University, Kaohsiung, Taiwan; 7 Department of Laboratory Medicine, Kaohsiung Medical University Hospital, Kaohsiung Medical University, Kaohsiung, Taiwan; 8 Department of Pediatrics, Kaohsiung Medical University Hospital, Kaohsiung Medical University, Kaohsiung, Taiwan; 9 Department of Biotechnology, College of Life Science, Kaohsiung Medical University, Kaohsiung, Taiwan; 10 Division of Hepatogastroenterology, Department of Internal Medicine, Chi-Mei Medical Center, Tainan, Taiwan; 11 College of Professional Studies, National Pingtung University of Science and Technology, Pingtung, Taiwan; Centers for Disease Control and Prevention, UNITED STATES

## Abstract

**Background:**

High dosage and longer duration of antiviral treatment has been suggested to treat cryoglobulinemia patients. We aimed to investigate the efficacy of antiviral treatment in cryoglobulinemia patients and analyze the associated factors of persistent cryoglobulinemia.

**Methods:**

Totally 148 patients after completion of anti-HCV treatment were enrolled in our study. Serum cryoglobulinemia precipitation was assessed and analyzed for the associated factors after antiviral therapy.

**Results:**

Fifty-one (34.5%) out of 148 patients were positive for serum cryoglobulinemia after completion of antiviral therapy. In multivariate analysis, advanced fibrosis (Odds Ratio [OR]– 4.13, 95% Confidence Interval [95% CI]– 1.53–11.17, p = 0.005) and platelet counts (OR-0.98, 95% CI– 0.97–0.99, p = 0.010) were independently and significantly associated with persistent cryoglobulinemia. The factors associated with the persistent cryoglobulinemia in SVR patients were advanced fibrosis (OR-1.93, 95% CI– 1.02–3.65, p = 0.041) and platelet count (OR-0.98, 95% CI– 0.96–0.99, p = 0.041) by multivariate analysis. Multivariate logistic regression analysis showed persistent (OR-4.83, 95% CI– 1.75–13.36, p = 0.002) was significantly associated with advanced fibrosis in patients with cryoglobulinemia follow up after antiviral therapy.

**Conclusions:**

The prevalence of the persistent cryoglobulinemia is 34.5% after completing antiviral therapy and it is associated with advanced fibrosis, also HCV clearance.

## Introduction

Hepatitis C virus (HCV) infection has been considered to be associated with extrahepatic manifestations and mixed cryoglobulinemia (MC) is one of the most common reported clinical extrahepatic symptoms [1]. MC is small and medium vessel vasculitis and considered as a B cell clonal expansion. The presence of the circulating serum cryoglobulinemia detected in 40–60% of HCV infected patients. However less than 10% of these patients may be present clinical vasculitis [2]. Although, ongoing HCV infection or the presence of anti-HCV antibody occurred in 90% of patients with MC and high concentration of HCV RNA, anti-HCV antibodies has been found in serum cryoprecipitate [3, 4], which means HCV is crucial for the development of cryoglobulinemia [5, 6]. Around 80% of the patients with cryoglobulinemic vasculitis and HCV infection were associated with older age, longer duration of HCV infection and kidney involvement [7, 8]. Also, cryoglobulinemia was associated with liver steatosis and advanced liver fibrosis in chronic hepatitis C (CHC) patients [9]. Careful monitoring of MC, MC vasculitis and chronic kidney manifestations during interferon based or interferon free antiviral treatment were suggested in CHC patients [10].

Treatment of HCV patients with cryoglobulinemia is indicated the progressive organ threatening disease is present. Patients without cryoglobulinemia symptoms only treated by antiviral treatment recommended by the guideline. In most cases, the successful treatment of HCV improved the cryoglobulinemia symptoms and complications [11, 12]. However, MC did not associate with antiviral treatment relapse and the patients with persistent cryoglobulinemia was related to more complicated diseases [13]. Because of the cost effectiveness, peginterferon and ribavirin treatment remains standard to treat HCV infected patients in most of the Asian countries [14] where direct antiviral agents (DAAs) are not available. However, interferon free DAA regimens cleared 50% of cryoglobulinemia and high response rate was reported [15, 16].

It has been suggested that the clearance of HCV by antiviral therapy was considered to reduce clinical manifestations of cryoglobulinemia and decreased the production of cryoglobulin [17]. However persistent cryoglobulinemia symptoms may occur after achieving sustained viral response (SVR) by pegylated interferon alpha plus ribavirin treatment [18] and suggested to treat high dose, longer duration of antiviral treatment for the patients with cryoglobulinemia [19]. Since Taiwan is an endemic area for HCV infection where peginterferon/ribavirin yields a higher SVR rate [20, 21], we have conducted this study to investigate the efficiency of antiviral therapy for cryoglobulinemia in CHC patients, as reflected by the disappearance of serum cryoprecipitate and analyze the factors associated with the persistent cryoprecipitate after anti-HCV therapy.

## Materials and methods

### Patients

**Fig 1:**
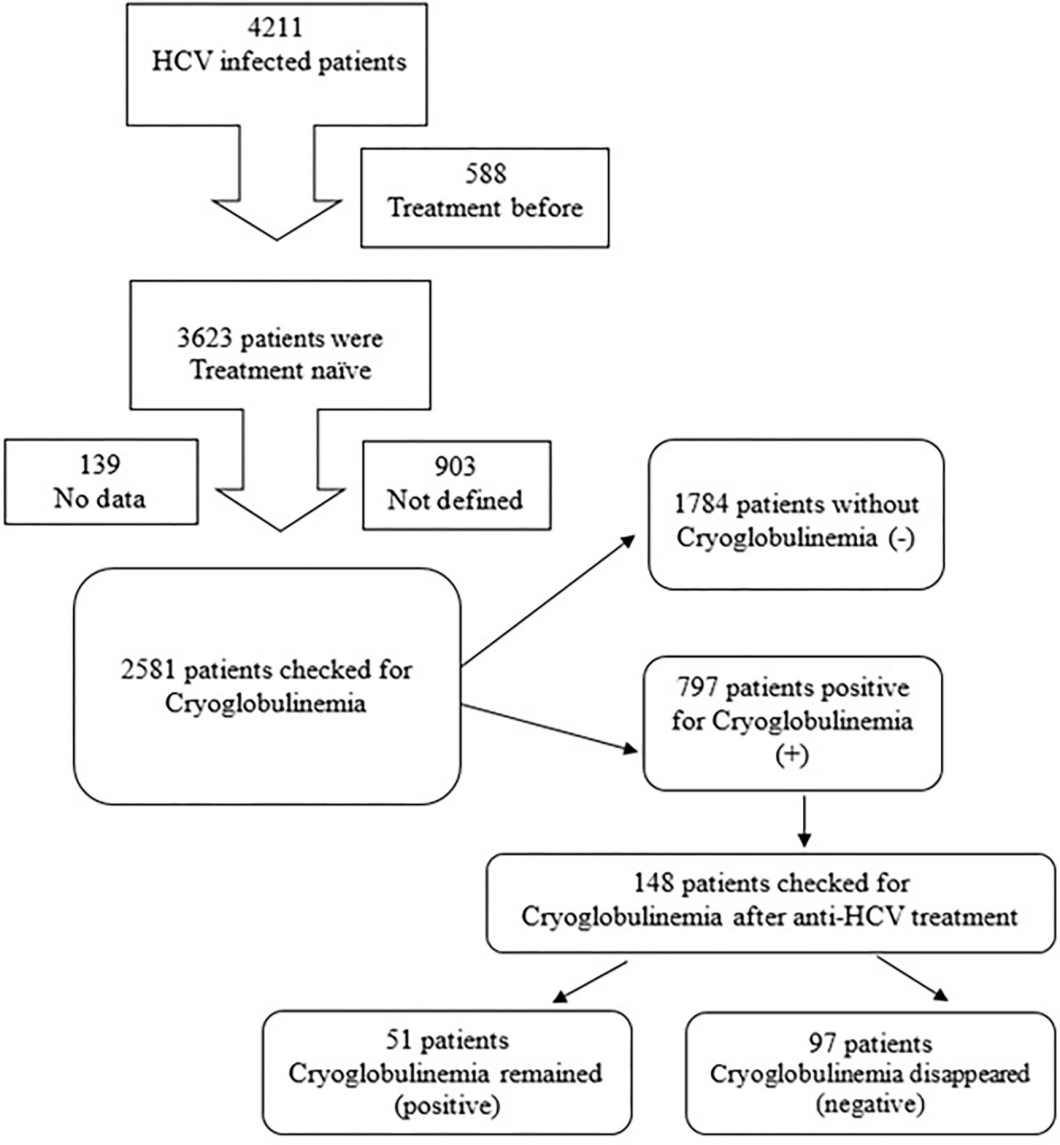
Study population.

We have conducted the multicenter study enrolling patients with CHC referred to Kaohsiung Medical University Hospital, a tertiary Medical Center and two regional hospitals from 2005 to 2016. There were 3,623 treatment naïve patients and 2,581 patients checked for serum cryoprecipitate and treated by pegylated interferon (PegIFN) /ribavirin treatment. One hundred and forty-eight patients who followed to check after completed antiviral therapy were enrolled in this study for the further analysis of the associated factors. The flowchart of study population is shown in Fig 1. CHC was defined as detectable serum HCV RNA (checked by COBAS AMPLICOR Hepatitis C virus test, version 2.0; Roche, Branchburg, NJ, USA (Detection limit– 50IU/mL)). We excluded patients with: a) co-infected patients with hepatitis B, D or human immunodeficiency virus, b) patients with connective tissue disorders, c) previous treatment for HCV infection and/or previous therapy with immunosuppressive drugs, d) past or current history of alcohol use, e) evidence of other liver disease (e.g., autoimmune hepatitis and primary biliary cirrhosis) and hepatocellular carcinoma.

### Treatment regimen

All patients had either PegIFN α-2a (Pegasys, Hoffmann-La Roche, Basal, Switzerland) 180 μg/week or Peg-IFN α-2b (PEG-Intron, Schering-Plough Inc., Kenilworth, NJ, USA) 1.5 μg/kg/week and additionally used weight-based ribavirin (1000 mg/d for <75 kg patients and 1200 mg/d for ≥75 kg respectively) for the antiviral treatment. All the patients were treated according to a response-guided therapy based on the HCV genotype, viral loads and viral response by the duration of 24–48 weeks which was the standard-of-care in the reimbursement guideline of Taiwan National Health Insurance [22]. The study design in concordance with ethical guidelines was approved by the Ethics Committee of Kaohsiung Medical University Hospital. All clinical investigations were conducted according to the principles laid down in the Declaration of Helsinki. Written informed consent was obtained from all subjects prior to enrollment in the study.

### Laboratory tests

#### Detection of cryoglobulinemia

Serum cryoprecipitation was detected by previously published method [23, 24] at the beginning and the end of the treatment. Briefly serum cryoprecipitate was defined the following procedure. 10–15 mL of blood was allowed to water bath at 37 ^0^C. Afterwards, up to 5 mL of serum was separated by centrifugation and placed under refrigeration (4 ^0^C). Serum was then observed for cryoprecipitation once a day for seven consecutive days. If a cryoprecipitate was noticed, tubes were re-incubated at 37 ^0^C for 30 min to verify dissolution of the cryoprecipitate. We confirmed the diagnosis of cryoglobulinemia by the presence of cryoprecipitate under refrigeration and its dissolution at 37 ^0^C in the serum of CHC proven patients in both the beginning and the end of the treatment.

Prior to initiation of treatment, general demographic characteristics and serum biochemical analyses using commercial tests were identified. These parameters included aspartate aminotransferase (AST), alanine aminotransferase (ALT), alpha fetoprotein (AFP) and platelets count. We used amplicons generated by the Amplicor HCV test using a commercially available assay (Line Probe assay, LIPA HCV, Innogenetics, Gent, Belgium) HCV genotypes were classified by the method proposed by Abbott RealTime HCV Genotype II, Abbott Molecular, Des Plaines IL, USA [25].

Sustained virological response (SVR) was defined as negative HCV RNA at 6-month after cessation of treatment. The four indexes of fibrosis (FIB4) was calculated to describe advanced fibrosis [26]. We used following formula to calculate FIB4:

$$FIB4=\left(age\;\left[years\right]\;x\;AST\;\left[U/L\right]\right)/\left(Platelet\;\left[10^{9}/l]\;x\;\surd ALT\;[U/L\right]\right)$$

Aspartate aminotransferase to Platelet Ratio Index (APRI) calculated by following formula [27].


$$\left(GOT\left[U/L\right]/GOT\left[Upper\;limit\;of\;normal\;range\right]\right)/Platelet\;\left[10^{9}/l\right]$$

### Histological evaluation

The liver biopsy was obtained for the patients before antiviral therapy and assessed according to the scoring system described by Knodell and Scheuer [28].

### Statistical analysis

Mean value and standard deviation were calculated in continuous variables. The continuous variables compared by Student’s T test, Chi-square (X^2^) or Fisher’s exact test were used for categorical variables. Univariate analysis and multivariate logistic regression analysis were performed to evaluate relationship between associated factors and persistent cryoglobulinemia. A P-value of less than 0.05 was considered to be statistically significant and based on two side hypotheses. All calculations were performed in the IBM SPSS Statistics for Windows, Version 20.0. Armonk, NY: IBM Corp.

## Results

### Factors associated with the persistent serum cryoglobulinemia after the antiviral therapy in patients with CHC

The demographic characteristics of 148 patients who was available to detect serum cryoglobulinemia after antiviral therapy are presented in Table 1. Fifty-one patients (34.5%) had persistent cryoglobulinemia after the treatment. Ninety-nine patients out of 148 were available for liver biopsy. Patients with persistent cryoglobulinemia were more likely to have advanced fibrosis (60%, *p* = 0.0001) and did not achieved SVR (47.1%, *p* = 0.038) compared to cryoglobulinemia disappeared patients. Other factors such as age, gender, HCV genotype and other HCV related diseases did not have any significant associations with the persistent cryoglobulinemia. From the laboratory parameters, low platelet count (130.6±51.9 10^9^/L, *p* = 0.001), higher fibrosis score (2.87±1.0, *p* = 0.0001) and higher FIB4 indexes (4.64±2.5, *p* = 0.019) were significantly associated with the persistent cryoglobulinemia. In multivariate analysis, advanced fibrosis (Odds Ratio [OR]– 4.13, 95% Confidence Interval [95% CI]– 1.53–11.17, *p* = 0.005) and platelet counts (OR-0.98, 95% CI– 0.97–0.99, *p* = 0.010) were independently and significantly associated with persistent cryoglobulinemia. PegIFN dose and treatment duration did not have any association (Table 1). However advanced fibrosis has more influenced to persistent cryoglobulinemia than viral clearance. If we adjusted FIB4 index instead of platelet count (Model 2), it was not associated with persistent cryoglobulinemia by multivariate analysis. Additionally, advanced fibrosis (OR-4.94, 95% CI– 1.90–12.8, *p* = 0.001) was the only significant factor associated with persistent cryoglobulinemia (Table 2).

**Table 1:** Baseline characteristics of patients with follow up after antiviral therapy.

	Univariate analysis				Multivariate analysis	
Characteristics	Total n-148	Persistence of	Disappearance of	P value	OR (95% CI)	P value
		Cryoglobulinemia (+) n-51 (34.5%)	Cryoglobulinemia (-) n-97 (65.5%)			
**Sex,** n (%)				0.890		
** Male**	65(43.9%)	22(43.1%)	43(44.3%)			
** Female**	83(56.1%)	29(56.9%)	54(55.7%)			
**Age** (mean±SD)	54.3±11.6	55.6±11.9	53.7±11.4	0.336		
**BMI** (kg/m^2^) (mean±SD)	24.9±3.4	25.1±3.6	24.8±3.4	0.577		
**HCV type,** n (%)				0.423		
** 1**	63(42.6%)	24(47.1%)	39(40.2%)			
** Non-1**	55(57.4%)	27(52.9%)	58(59.8%)			
**Diabetes,** n (%)				0.785		
** Yes**	176(15.5)	118(15.3)	58(15.9)			
** No**	959(84.5)	653(84.7)	306(84.1)			
**HCV RNA** (log IU/ml) (mean±SD)	5.3±2.0	5.6±1.5	5.1±2.2	0.146		
**Fibrosis stage,** n (%)				**0.0001**	4.13 (1.53–11.17)	**0.005**
** 0–2**	66(66.7%)	12(40%)	54(78.3%)			
** 3–4**	33(33.3%)	18(60%)	15(21.7%)			
**AST** (U/L) (mean±SD)	104.3±54.2	104.1±58.9	104.4±51.8	0.977		
**ALT** (U/L) (mean±SD)	145.1±84.8	130.6±76.8	152.7±88.1	0.132		
**AFP** (ng/mL) (mean±SD)	22.4±51.9	22.8±28.2	22.2±61.6	0.952		
**Platelet** (10^9^/L) (mean±SD)	153.1±59.3	130.6±51.9	164.9±59.8	**0.001**	0.98 (0.97–0.99)	**0.010**
**Viral response**				**0.038**	0.60 (0.22–1.65)	0.329
** SVR**	95(64.2%)	27(52.9%)	68(70.1%)			
** Non SVR**	53(35.8%)	24(47.1%)	29(29.9%)			
**FIB4**	3.90±2.8	4.64±2.5	3.51±2.8	**0.019**		
**PegIFN dose (mcg)**	3773.5±2024.3	3890.5±1946.6	3712.6±2071.1	0.616		
**PegIFN treatment duration**				0.218		
** <24 weeks**	108(75.5%)	34(69.4%)	74(78.7%)			
** >24 weeks**	35(24.5%)	15(30.6%)	20(21.3%)			

BMI–body mass index, AST—aspartate aminotransferase, ALT—alanine aminotransferase, AFP—alpha fetoprotein, FIB4 –four indexes of fibrosis, PegIFN–pegylated interferon, OR–Odds Ratio, 95% CI– 95% Confidence Interval. Bold values are statistically significant.

**Table 2:** Multiple logistic regression analysis for cryoglobulinemia persistence. Multivariate logistic regression model adjusted with SVR, fibrosis and FIB4.

Characteristics	Odds Ratio (95% Confidence Interval)	P value
SVR	0.51 (0.19–1.34)	0.173
Advanced fibrosis	4.94 (1.90–12.80)	**0.001**
FIB4	1.09 (0.93–1.26)	0.262

#### Factors associated with the persistent serum cryoglobulinemia after the antiviral therapy in patients who achieved SVR

The factors associated with the persistent cryoglobulinemia in SVR patients were advanced fibrosis (56.2%, *p* = 0.011) and low platelet count (137.3±56.3 10^9^/L, *p* = 0.012) respectively. Multivariate analysis revealed that the fibrosis stage (OR-1.93, 95% CI– 1.02–3.65, *p* = 0.041) and platelet count (OR-0.98, 95% CI– 0.96–0.99, *p* = 0.041) significantly associated with persistent cryoglobulinemia in SVR patients (Table 3).

**Table 3:** Univariate and multivariate analysis of the factors associated with SVR in patients with follow up after antiviral therapy.

	Univariate analysis				Multivariate analysis	
Characteristics	Total n-95	Persistence of	Disappearance of	P value	OR (95% CI)	P value
		Cryoglobulinemia (+) n-27 (28.4%)	Cryoglobulinemia (-) n-68 (71.6%)			
**Sex,** n (%)				0.818		
** Male**	44(46.3%)	12(44.4%)	32(47.1%)			
** Female**	51(53.7%)	15(55.6%)	36(52.9%)			
**Age** (mean±SD)	53.7±11.8	56.6±12.3	52.5±11.5	0.132		
**BMI** (kg/m^2^) (mean±SD)	24.5±3.2	24.5±3.2	24.5±3.2	0.970		
**HCV type,** n (%)				0.856		
** 1**	33(34.7%)	9(33.3%)	24(35.3%)			
** Non-1**	62(65.3%)	18(66.7%)	44(64.7%)			
**Diabetes,** n (%)				0.388		
** Yes**	13(13.7%)	5(18.5%)	8(11.8%)			
** No**	82(86.3%)	22(81.5%)	60(88.2%)			
**HCV RNA** (log IU/ml) (mean±SD)	4.8±2.1	5.3±1.4	4.7±2.3	0.147		
**AST** (U/L) (mean±SD)	104.6±55.8	102.8±62.3	105.3±53.4	0.842		
**ALT** (U/L) (mean±SD)	154.5±92.2	139.4±75.1	160.5±98.0	0.317		
**AFP** (ng/mL) (mean±SD)	21.5±61.2	15.3±19.0	24.1±71.8	0.560		
**Platelet (**10^9^/L) (mean±SD)	162.1±60.7	137.3±56.3	171.9±60.0	**0.012**	0.98 (0.96–0.99)	**0.041**
**Fibrosis stage,** n (%)				**0.011**	1.93 (1.02–3.65)	**0.041**
** 0–2**	45(69.2%)	7(43.8%)	38(77.6%)			
** 3–4**	20(30.8%)	9(56.2%)	11(22.4%)			
**HAI score** (mean±SD)	4.92±2.4	4.75±2.3	4.98±2.5	0.748		
**FIB4**	3.55±2.7	4.30±2.6	3.26±2.7	0.095		

BMI–body mass index, AST—aspartate aminotransferase, ALT—alanine aminotransferase, AFP—alpha fetoprotein, HAI–histology activity index, OR–Odds Ratio, 95% CI– 95% Confidence Interval. Bold values are statistically significant.

### Factors associated with advanced fibrosis in patients with follow up after the treatment

If we look at the related factors for advanced fibrosis, older age (56.2±11.2 years, *p* = 0.039), persistence of cryoglobulinemia (54.5%, *p*<0.0001) and lower platelet count (126.2±57.1 10^9^/L, *p* = 0.015) were associated by univariate analysis. Multivariate logistic regression analysis showed persistence of cryoglobulinemia (OR-4.83, 95% CI– 1.75–13.36, *p* = 0.002) were significantly associated with advanced fibrosis (Table 4).

**Table 4:** Univariate and multivariate analysis of the factors associated with advanced fibrosis in patients with follow up after antiviral therapy.

	Univariate analysis				Multivariate analysis	
Characteristics	Total n-99	F0-2 n-66	F3-4 n-33	P value	OR (95% CI)	P value
**Sex,** n (%)				0.666		
** Male**	42(42.4%)	29(43.9%)	13(39.4%)			
** Female**	57(57.6%)	37(56.1%)	20(60.6%)			
**Age** (mean±SD)	52.9±11.5	51.2±11.3	56.2±11.2	**0.039**	1.40 (0.99–1.08)	0.096
**BMI** (kg/m^2^) (mean±SD)	24.9±3.5	24.7±3.5	25.3±3.7	0.437		
**HCV type,** n (%)				0.247		
** 1**	40(40.4%)	24(36.4%)	16(48.5%)			
** Non-1**	59(59.6%)	42(63.6%)	17(51.5%)			
**Diabetes,** n (%)				0.451		
** Yes**	17(17.2%)	10(15.2%)	7(21.2%)			
** No**	82(82.8%)	56(84.8%)	26(78.8%)			
**HCV RNA** (log IU/ml) (mean±SD)	5.0±2.1	5.0±2.3	5.2±1.7	0.683		
**Cryoglobulinemia**				**0.0001**	4.83 (1.75–13.36)	**0.002**
** Disappeared**	69(69.7%)	54(81.8%)	15(45.5%)			
** Persisted**	30(30.3%)	12(18.2%)	18(54.5%)			
**AST** (U/L) (mean±SD)	109.8±49.7	108.2±51.0	113.1±47.7	0.646		
**ALT** (U/L) (mean±SD)	157.5±85.9	162.9±81.5	146.9±94.5	0.386		
**AFP** (ng/mL) (mean±SD)	25.6±61.1	18.2±23.6	38.7±96.4	0.253		
**Platelet** (10^9^/L) (mean±SD)	146.6±59.6	156.8±58.6	126.2±57.1	**0.015**	0.99 (0.98–1.006)	0.474
**Viral response**				0.454		
** SVR**	65(65.7%)	45(68.2%)	20(60.6%)			
** Non SVR**	34(34.3%)	21(31.8%)	13(39.4%)			
**FIB4**	4.13±3.0	3.71±3.2	4.97±2.4	0.050		

BMI–body mass index, AST—aspartate aminotransferase, ALT—alanine aminotransferase, AFP—alpha fetoprotein, FIB4 –four indexes of fibrosis, OR–Odds Ratio, 95% CI– 95% Confidence Interval. Bold values are statistically significant.

## Discussion

In this study we described the subset of HCV patients who completed pegylated interferon plus ribavirin therapy and experienced MC before and after therapy. Our results support that persistent cryoglobulienmia after antiviral therapy was associated with advanced liver fibrosis and influenced more than HCV clearance. Saadoun P et al. found an independent association between cryoglobulinemia and advanced liver fibrosis in patients with or without cryoglobulinemia vasculitis [9]. However, they analyzed the association of baseline cryoglobulinemia. In the present study we investigated the persistent cryoglobulinemia after completion of antiviral therapy. The association between hepatic necroinflammation and cryoglobulinemia was reported [29]. In the present study, low histology activity index was associated with SVR in CHC patients. Our previous study has shown the association between cryoglobulinemia and advanced fibrosis at the initiation of antiviral therapy in patients with or without liver biopsy [30]. It is unclear that the responsible mechanisms for the association between cryoglobulinemia and advanced fibrosis. However, host’s memory B cells expressed more in CHC and memory B cell CD86 related to the advanced liver fibrosis in CHC [31]. A higher frequency of T helper 1 cells were associated with activated memory B cells in HCV infection [32]. Additionally B cell homeostasis was related to the HCV induced MC because of the naïve B cell apoptosis [33].

Mahale P et al. described interferon based antiviral therapy induced SVR was reduced the risk of extrahepatic manifestations including MC [34]. Our study, to our knowledge, is the first study to investigate the persistent cryoprecipitation after antiviral therapy in CHC. The results showing the disappearance of the cryoblobulinemia after the anti-HCV therapy which supports the important role of the HCV RNA on the MC and the benefits of imporving this extrahepatic manifestation in anti-viral therapy. Stasi C et al. have investigated that the liver stiffness was reduced after combination of antiviral therapy plus rituximab and it is associated with B cell depletion [35]. Gragnani L et al. observed that the viral eradication allowed to reduce the MC syndromes and persistent clinical manifestations of MC after antiviral therapy was rare [36]. However multivariate analysis showed that the influence of advanced fibrosis was stronger than SVR in persistent cryoprecipitation in the present study.

Also previous study by Gragnani L et al. revealed that identified cryoglobulinemia has been an independent prognostic factor of nonresponse to the pegylated interferon plus ribavirin therapy [36]. In their report, the SVR rate was around 45.5% to 61.4% and 55% to 69.8% according to the intention-to-treat and the per protocol analysis, respectively. Also, several studies have investigated the therapeutic efficacy of PegIFN treatment and these studied were consistent for patients with HCV and cryoglobulinemia have lower rate of SVR compared to the patients without cryoglobulinemia [37–39]. Because of the characteristics of severe adverse events of Interferon based treatment, B cell depletion therapy (Rituximab) is necessary in addition to standard Interferon based antiviral therapy. In addition, standard Interferon based antiviral therapy plus Rituximab can be used for treat the patients with CHC and MC [40]. New direct acting antivirals (DAA) have dramatically high rate (>90%) of SVR, however in MC patients had still lower rate compared to CHC patients without cryoglobulinemia [41, 42]. Lauletta G et al. reported that DAAs had higher SVR rate, even in 12^th^ week of treatment, in previously treated patients. Also this study suggested longer follow up in CHC and MC patients to achieve complete response (viral and clinical) [43]. In the recent report of nine patients with HCV-associated cryoglobulinemic glomerulonephritis (CGN) by Obrișcă B, there are one "new-onset" CGN, six with persistent or worsening glomerulonephritis, and two with complete clinical response after achieving SVR12 for CHC without subsequent viral relapse by DAAs [44]. Further studies for the Asian patients seem mandatory.

Being the first study to investigate the persistent cryoprecipitation after antiviral therapy in CHC, our study, however, have some limitations. First, we did not have a chance to include the duration of HCV infection, which may relate to the cryoglobulinemia. Second, we were not be able to measure the quantitative value of cryoglobulin. Third, we did not have a possibility to assess the clinical manifestations of MC. Fourth, we have checked cryoglobulinemia after antiviral treatment in 148 patients. Also it is important to investigate in the future the skin lesions, joint pain, and peripheral neuropathy before and after treatment, especially in DAA treatment. Further investigation is needed to study cryoglobulinemia clearance in hepatitis c virus infected patients with direct acting antiviral treatment regimen.

In conclusion, our study shows an independent association between persistent cryoprecipitate and advanced fibrosis. Cryoglobulinemia in SVR achieved patients after completion of antiviral therapy is associated with the advanced liver fibrosis. Further investigation is needed to study the mechanism of cryoglobulinemia in advanced liver fibrosis.

## Supporting information

S1 Dataset (SAV)
